# Supplementary material for: A Web Survey to Evaluate the Thermal Stress Associated with Personal Protective Equipment among Healthcare Workers during the COVID-19 Pandemic in Italy
Source: Int J Environ Res Public Health. 2021 Apr 7;18(8):3861. doi: 10.3390/ijerph18083861 (PMC8067771; doi:10.3390/ijerph18083861)
Supplement: Supplementary file 1 [file ijerph-18-03861-s001.pdf]

THERMAL STRESS SURVEY DUE TO THE PPE USE DURING THE COVID-19 EMERGENCY - HEALTHCARE SECTOR (WORKLIMATE-Bric INAIL 2019 project)

The aim of this survey is to evaluate the impact of heat stress associated with the use of personal protective equipment (PPE) during the COVID-19 pandemic on healthcare workers.

The *ad hoc* survey was developed as part of the INAIL 2019 research activities within the WORKLIMATE project.

Filling out the questionnaire, anonymous and confidential, which takes about 15/20 minutes, does not include correct or incorrect answers, but only answers that reflect your personal perception and experience. The completeness of the answers is essential for the validity of the research.

Pursuant to the Regulation on the protection of natural persons with regard to the processing of personal data (EU Regulation 2016/679 - General Data Protection Regulation - GDPR - application from 25 May 2018), we inform you that the data transmitted by you will be collected and stored in an appropriate manner, analyzed in anonymous and aggregate form and will be used exclusively for scientific research purposes. The results of the research may be published in aggregate form, therefore your identity will remain anonymous.

Your contribution to this investigation is invaluable.

If you continue, you confirm your willingness to participate in our research and express your consent to the processing of the data provided.

*Required field

1- Date*

…………………

2- Place / Location where you work*

…………………………………..

3- Gender*

…………………………………

4- If you are a woman, are you pregnant?

-Yes
-No

5- Nationality*

-Italian
-EU-citizen
-non-EU citizen

6- Age*

……………………

7- Height*

……………………

8- Weight*

……………………

9- Kind of work*

-General practitioner
-Hospital doctor
-Dentist / Orthodontist
-Nurse / Pediatric Nurse
- Unlicensed assistive personnel
-Microbiologist / Biologist
-Physiotherapist
-Speech therapist
-Orthoptist/ assistant in Ophthalmology
-Podiatrist
-Psychiatric rehabilitation technician
-Neuro and psychomotor therapist of the developmental age
-Occupational therapist
-Dietitian
-Dental hygienist
-Audiology technician
-Hearing aid technician
-Technician of medical radiology, imaging and radiotherapy
-Neuro-pathophysiology technician
- Medical/biomedical laboratory technician
-Cardiocirculatory physiopathology and cardiovascular perfusion technician
-Orthopedic technician
-Healthcare assistant
- Technician for prevention in the environment and in the workplace

-Other

10- For physicians: area of specialization (medical area, surgical area; area of Clinical Services)

-Medicine
-Emergency-urgency medicine
-Geriatrics
-Sports and exercise medicine
-Thermal medicine
-Medical oncology
-Community medicine and primary care
-Allergology and clinical immunology
-Dermatology and Venereology
-Hematology
-Endocrinology and metabolic diseases
-Nutrition science
-Diseases of the digestive system
-Diseases of the cardiovascular system
-Diseases of the respiratory system
-Infectious and tropical diseases
-Nephrology
-Rheumatology
-Neurology
-Child Neuropsychiatry
-Psychiatry
-Pediatrics
-General surgery
-Pediatric surgery
-Plastic, reconstructive and aesthetic surgery
-Gynecology and Obstetrics
-Neurosurgery
-Orthopedics and traumatology
-Urology
-Maxillofacial surgery
-Ophthalmology
-Otolaryngology
-Cardiac surgery
-Thoracic surgery
-Vascular surgery
-Pathological anatomy
-Microbiology and Virology
-Clinical pathology and clinical biochemistry
-Radiodiagnostics
-Radiotherapy
-Nuclear medicine
-Anesthesia, resuscitation and intensive and pain therapy
-Audiology and phoniatrics
-Physical and rehabilitative medicine
-Clinical Pharmacology and Toxicology
-Medical genetics
-Hygiene and preventive medicine
-Occupational medicine

-Legal medicine
-Health Statistics and Biometrics

11- Department / place of employment. It is possible to select for this question more than an answer *

-Pre-triage under Tent
-Fever monitoring area
-Cleaning area
-COVID intensive care
-Non-COVID intensive care
-Anesthesia and intensive care
-Anesthesia, intensive care and analgesic therapy
-Pathological anatomy and histology
-Highly automated biochemistry
-Breast Unit
-Cardiac surgery
-Cardiology
-Pediatric cardiology
-Emergency medical service / Helicopter Rescue Area
-Center hub Hemophilia and congenital hemorrhagic diseases
-Emergency surgery
-Minimally invasive, regenerative and plastic surgery of the skin and appendages
-Maxillofacial surgery
-Plastic Surgery and Burn Center
-Breast surgery
-Thoracic surgery
-Vascular surgery
-General surgery
-Clinic and medical immunology
-Clinic and medical therapy
-Geriatric clinic
-Pediatric Clinic
-Pneumology outpatient clinic
-Oupatient cardiology clinic
-Day hospital Department
-Short stay - week surgery
-Dermatology
-Hematochemical diagnostics
-Department of Prevention
-Health management
-Hematology / Bone Marrow Transplant Center (Ctmo)
-Endocrinology and metabolic diseases
-Gastroenterology and digestive endoscopy
-Medical genetics
-Geriatrics
-Hygiene and Public Health
-Immunohematology and transfusion
-Immunogenetics of transplants
-Metabolic diseases and diabetology
-Infectious diseases and hepatology
-Occupational medicine and industrial toxicology

-Sleep medicine
-Internal Medicine
-Internal medicine for angiology and coagulation
-Internal medicine and critical long-term care
-Nuclear Medicine
-Rehabilitation medicine
-Microbiology
-Nephrology
-Neonatology
-Neurosurgery
-Neurology
-Neuroradiology
-Ophthalmology
-Odontostomatology
-Medical oncology
-Orthopedics
-Obstetrics and Gynecology
-Otolaryngology and otoneurosurgery
-Pediatrics and oncohematology
-General and Emergency Pediatrics
-Pulmonology and thoracic endoscopy
-Emergency-urgency center
-First aid and emergency medicine
-Psychiatry
-Radiology
-Radiotherapy
-Local network of palliative care
-Radiological Sciences
-Toxicology
-Urology
-Virology
-Dental practice
-Analysis / microbiology laboratory

12- During the hot period (between May and September), do you avoid eating on fast days for personal (religious / ethical / nutritional ...) reasons? *

0 never 1 2 3 4 5 always

13- How do you judge your work effort on average?

-At rest
-Lightweight
-Moderate
-High
-Very high

14- How would you judge the thermal environment in which you generally work? *

-Very cold
-Cold
-Slightly cold

-Neutral
-Slightly hot
-Hot
-Very hot

15- In your work shift, how many hours do you usually wear the PPE listed in the table below in this COVID-19 pandemic ? *

	0 hours	1 to 3 hours	4 to 6 hours	Over 6 hours
N95 mask or equivalent (FFP2)				
FFP3 mask				
Surgical mask				
Gloves (one pair)				
Gloves (two overlapping pairs)				
Disposable gown				
Normal gown				
Disposable apron				
Glasses				
Visor				
Headgear				
Closed boots or work shoes				
Shoes covers				
Sanitary clogs				

16- How many days per week do you use PPE during your work? *

1 2 3 4 5 6 7

17- How long does it take to wear PPE at the start of the work shift? (in minutes) *

18- Do you work mainly in an air-conditioned environment? *

-Yes
-No

19- If you have taken heat-related sick leave in the last 3 months, can you indicate the number of days?

20- Is there a company procedure that allows you to remove PPE during work breaks? *

-Yes
-No

21- If "YES", when? It is possible to select more than one answer for this question

-in the middle of the day for lunch
-when I go to the toilet
-after each visit
-whenever I need to

22- Is there a dedicated rest area in the workplace? *

-Yes
-No

23- How do you try to reduce heat stress when using PPE? It is possible to select more than one answer for this question *

-I often drink water
-I drink ice cold drinks
-I take breaks whenever possible
-I try to dress in light clothing
-Breathing techniques
-I prefer ventilated and cool environments

24- What symptoms do you generally perceive when you work wearing PPE? It is possible to select more than one answer for this question *

-Thirst
-Excessive sweating
-Fatigue
-Headache
-Difficulty concentrating
-Skin reaction
-General discomfort

25- Do you feel a difference in temperature between the parts of the body covered and not covered by PPE when you work? *

-Yes
-No

26- What is your thermal sensation when you dress PPE during work activities? *

-Very cold
-Cold
-Slightly cold
-Neutral
-Slightly hot
-Hot
-Very hot

27- What is your thermal sensation in the parts of your body covered by PPE when you work?

	Very cold	Cold	Slightly cold	Neutral	Slightly hot	Hot	Very hot
Upper part of the face							
Lower part of the face							
Neck							
Hands							
Back							
Chest							
Armpits							
Legs							
Whole body							

Tell us how much you agree with the following statements:

28- Heat stress can affect my work productivity *

1 (completely disagree) 2 3 4 5 (completely agree)

29- Heat stress can impair my reasoning *

1 (completely disagree) 2 3 4 5 (completely agree)

30- Heat stress can affect my physical health *

1 (completely disagree) 2 3 4 5 (completely agree)

31- Heat stress can negatively affect my psychological state *

1 (completely disagree) 2 3 4 5 (completely agree)

32- Heat stress can negatively affect my emotions *

1 (completely disagree) 2 3 4 5 (completely agree)

33- Heat stress can negatively affect my commitment at work *

1 (completely disagree) 2 3 4 5 (completely agree)

34- Keeping fit will improve my heat tolerance *

1 (completely disagree) 2 3 4 5 (completely agree)

35- A good hydration before the work shift will improve my heat tolerance *

1 (completely disagree) 2 3 4 5 (completely agree)

36- Adequate rest between shifts will improve my heat tolerance *

1 (completely disagree) 2 3 4 5 (completely agree)

37- Wearing PPE is uncomfortable for me *

1 (completely disagree) 2 3 4 5 (completely agree)

38- I'm too busy when I work and consequently I can't take breaks *

1 (completely disagree) 2 3 4 5 (completely agree)

39- My work productivity is reduced when I wear PPE *

1 (completely disagree) 2 3 4 5 (completely agree)

40- It is important to keep hydrated during the work shift *

1 (completely disagree) 2 3 4 5 (completely agree)

41- It's uncomfortable to take breaks to rehydrate *

1 (completely disagree) 2 3 4 5 (completely agree)

42- I avoid taking breaks to not remove and put on the PPE again *

1 (completely disagree) 2 3 4 5 (completely agree)

43- I avoid drinking and eating so as to reduce breaks to use the toilet *

1 (completely disagree) 2 3 4 5 (completely agree)

44- I avoid taking breaks to reduce the risk of getting infected *

1 (completely disagree) 2 3 4 5 (completely agree)

45- Slush drinks improve my tolerance to heat *

1 (completely disagree) 2 3 4 5 (completely agree)

46- The PPE I wear prevent the evaporation of sweat *

1 (completely disagree) 2 3 4 5 (completely agree)

Thank you for taking part in the survey, if you prefer you can leave comments and / or suggestions
